# Single-cell multi-omics for precision cardiovascular and longevity medicine: from methods to clinical translation

**DOI:** 10.3389/fragi.2025.1656727

**Published:** 2025-11-27

**Authors:** Thiago Guimarães Osório, Estefania Pavesi, Khalil Abou El-Ardat, Needa Qureshi, Leanne Cassidy, Terrence Lee St. John, Nicole Sirotin, Bartlomiej Piechowski-Jozwiak

**Affiliations:** 1 Institute for Healthier Living Abu Dhabi LLC, Abu Dhabi, United Arab Emirates; 2 Singleron Biotechnologies GmbH, Cologne, Germany

**Keywords:** single-cell multi-omics, precision medicine & genomics, immune aging, cardiovascular aging, geroscience, longevity

## Abstract

**Background:**

Single-cell multi-omics (SCMO) technologies simultaneously profile multiple molecular layers (e.g., DNA, RNA, proteins) within individual cells. Unlike traditional bulk analyses that average signals across thousands of cells, SCMO captures the unique molecular characteristics of each cell, potentially transforming our understanding of disease pathogenesis and clinical management. Despite its promise, SCMO methodologies remain complex and difficult for clinicians to interpret and utilize effectively.

**Methods:**

We conducted a narrative review specifically tailored to clinicians, summarizing key SCMO methodologies, recent discoveries, and their translational relevance for cardiovascular and aging-related diseases. Our goal was to simplify complex SCMO concepts, highlight practical clinical insights, clarify methodological details in accessible terms, and openly discuss barriers currently preventing routine clinical implementation.

**Results:**

SCMO techniques have identified clinically relevant cellular heterogeneity within diseases such as atherosclerosis and heart failure, uncovering subpopulations linked to disease severity and potential therapeutic targets. Notably, SCMO studies revealed specific inflammatory immune subsets in unstable plaques, pathogenic fibroblast populations driving cardiac fibrosis, and distinct immune profiles associated with aging and longevity. Early clinical trials integrating SCMO demonstrate feasibility in oncology and cardiology, and prototype clinical assays (e.g., single-cell “liquid biopsies”) are emerging. These advances are yielding predictive biomarkers and guiding personalized and preventive applications.

**Conclusion:**

SCMO is rapidly evolving, offering unprecedented precision in diagnostics and personalized therapeutics by pinpointing disease-driving cells and molecular pathways. However, significant hurdles including high costs, technical complexity, and analytical challenges currently limit immediate clinical application. To the best of our knowledge, as of 2025, FDA authorization for single-cell diagnostics is limited to established technologies like flow cytometry, while next-generation multi-omic platforms remain confined to research use. This manuscript is explicitly designed to help clinicians navigate the complexity of SCMO, providing clear, digestible explanations of its methodologies and emphasizing how these tools might practically benefit patient care. Clinicians should remain cautiously optimistic, viewing SCMO as a complementary, specialized tool. Continued technological and methodological advances suggest SCMO will become increasingly integral to precision medicine.

## Introduction

Single-Cell Multi-Omics (SCMO) refers to a suite of cutting-edge techniques that profile multiple molecular layers (e.g., genome, transcriptome, epigenome, proteome) within individual cells (Hu et al., 2018; Tang et al., 2019; Wu et al., 2024). Unlike traditional “bulk” analysis techniques such as quantitative PCR, microarray analysis, bulk RNA sequencing (Heid et al., 1996), which average signals across thousands of cells, SCMO preserves cellular heterogeneity, the differences between individual cells that often determine disease behavior.

Over the past decade, single-cell technologies, initially led by single-cell RNA sequencing, have transformed our understanding of biology by uncovering rare cell types and dynamic cell-state changes that were previously invisible in bulk data (Tang et al., 2009; Jaitin et al., 1979; Ramsköld et al., 2012; Hashim et al., 2012). Building on this, SCMO integrates diverse data types from the same cell (for example, linking a cell’s gene expression with its chromatin accessibility or surface protein markers), yielding a richer, multidimensional view of cellular function. This level of resolution facilitates the mapping of tissue ecosystems in both health and disease, offering a path to identify pathogenic subpopulations and therapeutic targets with unprecedented precision.

Cardiovascular diseases (CVD) of aging, such as atherosclerosis, myocardial infarction (MI), and heart failure (HF), are prime targets for single-cell precision medicine (Kuppe et al., 2022; Wirka et al., 2019; Mosquera et al., 2023). Age is the dominant risk factor for these conditions (Khan et al., 2024; North and Sinclair, 2012). The emerging field of geroscience emphasizes that fundamental aging processes (e.g., chronic inflammation, cellular senescence) underlie many chronic diseases. Indeed, age-related clonal expansions of immune cells (clonal hematopoiesis) and “inflammaging” contribute to CVD risk by boosting inflammatory pathways (Liberale et al., 2022; Jaiswal et al., 2017). Traditional clinical tools have limited ability to capture this complexity, like C-reactive protein provide only a crude signal. SCMO offers a new paradigm: by resolving the contributions of individual cell subsets, it can identify pathogenic cell types and molecular circuits driving disease in older adults. In turn, these insights pave the way for truly precision medicine interventions: targeted drugs against key cellular players, cell-based therapies, or refined biomarker-driven patient stratification.

Notably, single-cell studies have already generated clinically relevant findings in CVD that could never be established with bulk techniques (Tang et al., 2019; Wang and Jin, 2024). For example, cardiac single-cell atlases have defined numerous distinct cell types and their interactions, enabling researchers to link specific cell subtypes to disease outcomes (Paik et al., 2020). The increase of single-cell studies (in cardiovascular research alone, publications have increased exponentially since 2015) signals a paradigm shift (Dai and Nomura, 2021). For clinicians across specialties, the relevance is clear: SCMO is illuminating disease mechanisms, revealing novel biomarkers and drug targets, and paving the way for precision medicine approaches tailored to the cellular makeup of each patient’s disease (Hu et al., 2018; Paik et al., 2020; Xiong et al., 2023). This review provides an accessible overview of SCMO, emphasizing clinical implications rather than focusing only on technical details.

This review focuses on cardiovascular aging, highlighting how single-cell multi-omics is illuminating the pathophysiology of atherosclerosis, myocardial infarction, and heart failure in the context of the aging process. We present key SCMO findings in these diseases, organized as clinical narratives that connect molecular mechanism to potential therapy. Furthermore, we provide a roadmap for translating these discoveries into clinical applications, critically addressing the essential considerations of reproducibility, validation, and regulatory approval. Throughout this manuscript, the emphasis is placed on clinically relevant insights, aiming to equip clinicians and researchers with an accessible yet deep understanding of how single-cell multi-omics can drive the future of precision cardiovascular medicine.

### Single-cell multi-omics methodologies

To establish a foundation for understanding the clinical applications of SCMO, this section provides an overview of the key technologies. Each modality possesses distinct strengths and limitations, particularly in the context of analyzing human clinical samples, which are often challenging to procure and process. The evolution of these technologies reflects a continuous effort to overcome practical barriers, moving from tools requiring fresh, pristine tissue toward more robust methods compatible with the realities of clinical biobanking.

### Single-cell and single-nucleus RNA sequencing: capturing transcriptional heterogeneity

Single-cell (scRNA-seq) and single-nucleus RNA sequencing (snRNA-seq) are foundational tools in SCMO, providing insights into gene expression at single-cell resolution. While scRNA-seq captures full transcriptomes from intact cells, snRNA-seq profiles nuclear RNA, offering advantages in fibrotic, archived, or otherwise dissociation-resistant tissues such as myocardium or atherosclerotic plaques.

These technologies have proven instrumental in aging research. A landmark study applied scRNA-seq to peripheral blood mononuclear cells (PBMCs) from centenarians and their offspring, identifying a transcriptional signature marked by upregulation of ribosomal genes and dampened pro-inflammatory pathways (Zhu et al., 2023). This balance correlated with a youthful immune phenotype.

Model organisms have provided mechanistic insights: one study used single-cell transcriptomics in aging yeast and found early divergence in oxidative stress responses and transcriptional noise, suggesting conserved early markers of cellular aging (Wang et al., 2022).

Technically, sc/snRNA-seq requires careful cell or nuclei isolation, barcoding, cDNA amplification, and computational analysis. Platforms like Singleron’s SCOPE™ chip optimize microfluidic workflows for complex tissues, minimizing batch effects and enhancing throughput. Established players in the field, such as 10x Genomics, also offer widely adopted platforms that have set benchmarks for scalability and data quality in single-cell sequencing.

Recent advances, such as SMART-seq3 and sci-RNA-seq3, now offer improved sensitivity and the ability to trace splicing dynamics, RNA velocity, and cellular differentiation trajectories. These tools are invaluable in tracking senescence, stress responses, and fate commitment in aging or disease-affected cardiovascular tissues (Hagemann-Jensen et al., 2020). SMART-seq3 is commercially available through companies like Takara Bio, while sci-RNA-seq3, primarily developed in academic settings, is increasingly supported by platforms such as Parse Biosciences for scalable combinatorial indexing solutions.

### Single-cell metabolomics: charting biochemical states in aging and disease

While transcriptomics offers a snapshot of gene activity, metabolomics reflects the cell’s biochemical function in real time. Single-cell metabolomics, especially via mass spectrometry (MS), has become increasingly powerful. Techniques like MALDI-MS, SIMS, and capillary electrophoresis-MS allow detection of metabolites at femtomolar concentrations with spatial resolution.

Notably, advanced platforms now allow single-cell metabolite profiling in the context of aging and senescence. A recent study introduced a microfluidic SlipChip system combined with surface-enhanced Raman spectroscopy (SlipChip–SERS) to non-destructively profile intracellular metabolites in individual senescent cells (Liu et al., 2024). This approach identified elevated spermine, a polyamine associated with chromatin structure and DNA protection, as a potential biomarker of senescence.

Complementing this, researchers combined live-cell imaging with single-cell MS to link metabolic heterogeneity to senescence phenotypes. By using optical markers such as reactive oxygen species (ROS) and senescence-associated β-galactosidase (SA-β-gal), they correlated metabolite signatures, especially related to NAD^+^ metabolism and glutathione homeostasis, with distinct cellular aging states (Wang Z. et al., 2025).

Investigators futher developed a metabolomic atlas of aged murine hematopoietic stem cells (HSCs), identifying uridine as a rejuvenating metabolite. Interestingly, their data showed that metabolic reprogramming, particularly in nucleotide biosynthesis, preceded and influenced downstream transcriptomic shifts (Zeng et al., 2024).

However, technical hurdles remain. Ion suppression, metabolite degradation, and data normalization continue to limit sensitivity. Computational approaches such as probabilistic quotient normalization and baseline correction are increasingly adopted to minimize inter-sample variability (Sun and Xia, 2024).

When integrated with transcriptomic and spatial data, single-cell metabolomics provides a powerful systems-level view of cellular senescence, immune dysfunction, and fibrotic remodeling during aging and cardiovascular disease.

### CITE-seq: linking transcription and surface proteome

Cellular Indexing of Transcriptomes and Epitopes by Sequencing (CITE-seq) expands the power of single-cell RNA sequencing by simultaneously measuring surface protein expression using oligonucleotide-tagged antibodies. This dual-modality approach provides a more complete picture of cell state, bridging the gap between transcript abundance and protein phenotype.

A landmark study applied CITE-seq to human atherosclerotic plaques, profiling immune cells at single-cell resolution (Fernandez et al., 2019). The integration of transcriptomic and proteomic data enabled fine-grained discrimination of macrophage subtypes, including TREM2^+^ foam cells, activated NK cells, and inflammatory myeloid populations. This combined approach provided significantly improved cell classification and functional annotation, highlighting the value of surface protein information in resolving immune heterogeneity in vascular disease (Bashore et al., 2023; Depuydt et al., 2020).

Another study in the context of aging and cardiovascular disease used CITE-seq on peripheral blood immune cells to reveal age-associated changes in T cell and monocyte subsets that were not detectable at the transcript level alone. Specifically, they identified an expanded population of CD16^+^ monocytes and exhausted CD8^+^ T cells in aged individuals, characterized by protein markers such as CD57 and PD-1 (Wang Y. et al., 2025; Terekhova et al., 2023; Huang et al., 2021).

From a technical standpoint, successful CITE-seq experiments require high-quality antibody panels, careful titration to avoid saturation, and bioinformatic deconvolution of antibody-derived tag data. Despite these complexities, its integration with transcriptomic and metabolic data enables multi-dimensional profiling of immune aging and vascular inflammation.

### Spatial omics: embedding single-cell data in tissue architecture

Spatial transcriptomics enables the mapping of gene expression within the native architecture of tissues, providing essential context for understanding complex biological processes such as fibrosis, inflammation, and tissue remodeling. Techniques like Slide-seqV2 and MERFISH (Multiplexed Error-Robust Fluorescence *in Situ* Hybridization) have dramatically increased resolution and throughput, allowing the study of thousands of transcripts in spatially organized cell populations.

In aging and cardiovascular research, spatial transcriptomics has revealed regional gene expression differences that are not detectable in dissociated single-cell data. For example, using spatial transcriptomics to generate a cell atlas of the developing and adult human heart, identifying distinct fibroblast and endothelial subtypes localized to specific anatomical regions (Asp et al., 2019). These spatial signatures provided insights into disease-prone areas and age-related tissue changes.

Similarly, researchers applied spatial transcriptomics to human heart biopsies and discovered spatially organized fibroblast activation patterns associated with age and fibrotic remodeling (Kuppe et al., 2022). These patterns correlated with histological features of heart failure and were enriched in pro-fibrotic gene signatures.

Newer platforms now combine transcriptomics with spatial proteomics (e.g., CODEX) or metabolomics (e.g., MALDI imaging), offering multi-omic integration *in situ*. When aligned with scRNA-seq data, these methods enhance cell-type resolution and uncover spatially regulated pathways in tissue aging.

### Metallomics: mapping essential elements in aging tissues

Metallomics investigates the distribution and dynamics of essential metal ions, such as iron (Fe), zinc, copper, and calcium, which play crucial roles in maintaining redox balance, facilitating enzymatic functions, and regulating mitochondrial metabolism. Disruption of these elements is increasingly linked to aging-related processes, including ferroptosis, fibrosis, and inflammation (López-Otín et al., 2023).

State-of-the-art single-cell and high-resolution methods, such as SC-ICP-MS (single-cell inductively coupled plasma mass spectrometry), laser ablation ICP-MS, synchrotron X-ray fluorescence, and mass cytometry, now allow spatially resolved measurement of elemental concentrations, often approaching single-cell resolution. One group comprehensively reviewed SC-ICP-MS, LA-ICP-MS, nanoSIMS, and SXRF applications for mapping intracellular metal distribution at single-cell levels (Jiménez-Lamana et al., 2018).

On the tissue level, a recent Nature Metabolism study found that iron accumulation is a hallmark of cellular senescence and fibrosis: senescent cells accumulate ferritin-bound iron, elevating labile intracellular Fe^2+^, which in turn fuels reactive oxygen species generation and stimulates senescence-associated secretory phenotype pathways (Maus et al., 2023).

Although true single-cell metallomic studies in aging are still emerging, these spatial and elemental data complement transcriptomic and proteomic profiles by revealing chemical and redox landscapes invisible to nucleic acid–based methods. As SC-ICP-MS and metal-tagged immunophenotyping methods mature, they are likely to become key tools in single-cell multi-omic studies of aging and cardiovascular disease.

## Cardiovascular applications of SCMO

### Atherosclerosis and coronary artery disease

Atherosclerosis is a chronic inflammatory disease marked by lipid-rich plaques in arterial walls, and it is a prime example of an age-associated pathology (Libby et al., 2002). Its most devastating clinical outcomes, such as myocardial infarction and stroke, are often precipitated by plaque rupture, an event influenced by the lesion’s cellular composition and inflammatory activity (Lin et al., 2024; Cole et al., 2018; Cochain et al., 2018). SCMO approaches have been instrumental in unravelling this cellular heterogeneity and identifying cell subpopulations linked to disease severity. For instance, macrophages with similar morphology can differ substantially in transcriptional programs: some display pro-inflammatory, plaque-destabilizing signatures, while others contribute to resolution and stability (Lin et al., 2024). Capturing these differences is clinically important. It may explain why some plaques remain stable while others rupture, and such nuances were impossible to discern with bulk tissue analysis. SCMO thus provides a kind of cellular “histo-pathology” at high resolution, parsing the good actors from the bad within the immune infiltrate. In addition to immune cells, vascular smooth muscle cell (SMC) phenotypic modulation has also emerged as a key driver of plaque behavior. An integrated single-cell meta-analysis recently identified SMC subsets with fibrochondrogenic features that correlate with advanced plaque progression and calcification (Mosquera et al., 2023). Similarly, another scRNA-seq study discovered a unique multi-lineage SMC subpopulation in human coronary plaques that interacts with macrophages via TNF and ANGPTL signaling to shape the plaque’s inflammatory microenvironment (Ye et al., 2025).

Crucially, single-cell studies are not just cataloguing cell types; they are correlating them with clinical endpoints and suggesting actionable targets. Single-cell analyses of human atherosclerotic plaques have identified 28 distinct immune cell subpopulations, including specific T-cell and macrophage phenotypes associated with worse outcomes. Notably, a pro-inflammatory subset of CD_4_^+^ T cells lacking CD28 was found to predict a poor prognosis in patients. These cells were enriched in advanced, high-risk plaques. Likewise, researchers discovered a dysfunctional “foamy” macrophage subset that localizes to hypoxic plaque cores and exhibits impaired cholesterol handling but high pro-angiogenic activity. Intriguingly, this macrophage subtype was regulated by mast cell-derived CSF1 and was far more abundant in plaques that had ruptured or hemorrhaged, linking it to unstable disease (Xiong et al., 2023). Such findings have immediate translational appeal: these cell populations (or their secreted factors) could serve as biomarkers to identify patients with dangerous plaque biology, or as targets for tailored immunotherapies. Indeed, the limited success of broad anti-inflammatory therapies in trials (e.g., with colchicine therapy or systemic IL-1β) may be due to a one-size-fits-all approach neglecting immune heterogeneity (Nidorf et al., 2020; Ridker et al., 2017; R et al., 2018). A more precise strategy would be to target the pathogenic subsets while sparing immune functions necessary for host defense. Single-cell profiling makes this feasible by delineating which immune cells to hit or spare. Additionally, SCMO has been applied beyond the plaque itself, for example, to circulating blood, liver, and gut in atherosclerotic models, revealing system-wide changes and potential peripheral biomarkers of early disease (Lin et al., 2024).

The long-term vision is that, in the future, a patient’s blood might be analyzed at single-cell resolution to detect the emergence of pro-atherogenic immune cell signatures before a plaque ever causes symptoms, enabling preemptive interventions. While this remains aspirational, the current trajectory suggests that diagnostic, prognostic, and therapeutic insights from SCMO in atherosclerosis are steadily transitioning from bench to bedside.

### Myocardial infarction: injury and repair response

Myocardial infarction continues to be one of the foremost causes of death worldwide (Wong, 2014). Age is a major predictor of worse post-MI outcomes, including heart failure and death. Besides age, the cellular composition of atherosclerotic plaques is increasingly recognized as a key factor in MI risk. A scRNA-seq meta-analysis identified phenotypically modulated SMC subsets in plaques associated with higher MI and increased incidence of coronary disease and calcification (Mosquera et al., 2023). Despite notable progress in acute-phase management, the limited understanding of post-infarction remodeling still impedes efforts to reduce late-stage mortality (Niccoli et al., 2019). Acute MI triggers an abrupt immune and reparative response in the heart, and SCMO has been essential in dissecting this highly time-dependent cellular reaction. Within an infarcted myocardium, multiple cell types (neutrophils, monocyte-derived macrophages, lymphocytes, fibroblasts, endothelial cells, cardiomyocytes, etc.) coordinate to clear necrotic tissue and form a scar. If this healing process is dysregulated, however, it can contribute to adverse remodeling and complications like heart failure or even cardiac rupture (Psarras et al., 2019; Frangogiannis, 2014; Epelman et al., 2015). Traditional cardiac biomarkers (e.g., white blood cell counts or CRP) only crudely reflect this process. In contrast, single-cell transcriptomic analyses have mapped the precise gene programs activated in each cell subset during MI and identified new therapeutic opportunities (Pekayvaz et al., 2024; Molenaar et al., 2021). For example, an early single-cell study profiled leukocytes from mouse hearts after MI and discovered a distinct subpopulation of cardiac macrophages that mounted a robust type I interferon response, driven by the transcription factor interferon regulatory factor 3 (IRF3). This interferon-producing macrophage subset was found to exacerbate post-MI inflammation. Notably, when researchers blocked the IRF3 interferon pathway, mice exhibited lower levels of inflammatory cytokines and chemokines in the heart and showed improved cardiac function after MI (King et al., 2017). This finding directly links a single-cell observation to a therapeutic concept, selective immunomodulation of deleterious macrophage activation to improve healing. Similarly, single-cell analyses revealed that regulatory T-cells (Tregs) accumulate in the injured myocardium and play an unexpected beneficial role (Xia et al., 2020). These heart-infiltrating Tregs were largely recruited from circulating Tregs and exhibited a unique T-cell receptor repertoire and gene signature, including high expression of SPARC (a matricellular protein). Functionally, these Tregs were shown to promote collagen deposition and scar organization, protecting against cardiac rupture in mouse MI models. Blocking SPARC in Tregs impaired this protective effect, suggesting that augmenting Treg responses or their key factors might fortify infarct stability (Xia et al., 2020; Vafadarnejad et al., 2020; Wang et al., 2023).

Beyond the immune system, SCMO has shed light on the behavior of cardiac fibroblasts, the primary drivers of scar formation. Single-cell sequencing of infarcted mouse hearts identified previously unrecognized fibroblast subsets that emerge only after injury. For instance, a scRNA-seq study identified a subset of activated fibroblasts uniquely present in injured hearts, characterized by high expression of CKAP4, a receptor protein whose function in the heart was previously unknown (Gladka et al., 2018). This subset’s gene profile suggested a particularly profibrotic role. When CKAP4 was experimentally inhibited in fibroblasts, the cells exhibited exaggerated activation in response to TGF-β, suggesting that CKAP4 may generally act as a brake on fibrotic activation. This raises notion that modulating CKAP4 or similar receptors could fine-tune fibroblast activity after MI, potentially enhancing scar quality or limiting excessive fibrosis (Gladka et al., 2018). Other single-cell investigations have mapped intercellular crosstalk in the post-MI heart: for example, injured cardiomyocytes were found to secrete β2-microglobulin (B2M), which neighbouring fibroblasts sensed via corresponding receptors, inducing those fibroblasts to transform into myofibroblasts (contractile, scar-forming cells). Blocking B2M signaling blunted this myofibroblast conversion *in vitro*, suggesting another targetable pathway to mitigate pathological remodeling (Molenaar et al., 2021). Meanwhile, single-cell data have identified anti-fibrotic fibroblast phenotypes as well. One study identified a fibroblast subpopulation expressing Wif1 that emerged after MI with an “anti-WNT/anti-TGFβ” profile, potentially limiting fibrosis and promoting angiogenesis in border zones (Farbehi et al., 2019). Although these findings are in animal models, they underscore a future in which clinicians could manipulate not just fibrosis in general, but specific fibroblast sub-lineages or their signaling axes to optimize wound healing post-MI. This targeted approach could help to improve outcomes after MI, one of the most prevalent non-communicable diseases worldwide.

Integrated SCMO in MI has yielded actionable insights. A recent study in human hearts combined single-nucleus RNA-seq and single-nucleus ATAC-seq (chromatin accessibility profiling) on tissue from patients with prior MIs (Kuppe et al., 2022). By correlating gene expression changes with upstream chromatin markers, the researchers identified RUNX1, a transcription factor, as a candidate master regulator that drives the differentiation of fibroblasts into myofibroblasts following infarction. They observed increased RUNX1 expression and increased accessibility of RUNX1 binding motifs in the nuclei of fibroblasts from post-MI hearts, indicating the active engagement of RUNX1-controlled genetic programs. Follow-up experiments confirmed that RUNX1 amplifies TGF-β signaling in these cells, promoting scar-forming myofibroblast phenotypes. This type of multi-omics evidence elevates RUNX1 as a potential therapeutic target; inhibiting RUNX1 activity may reduce maladaptive fibrosis after MI and improve cardiac repair. Such insights, gleaned from actual patient tissues via SCMO, exemplify how multi-omics can pinpoint key molecular “levers” of disease, even in a complex post-infarction environment, and thereby inform the development of targeted therapies (e.g., a RUNX1 inhibitor to prevent stiff scar and heart failure).

In summary, SCMO studies in MI are providing a cellular roadmap of the acute injury response, highlighting which cell types and signals to encourage and which to dampen. Clinically, this could translate into better risk stratification (e.g., measuring a patient’s proportion of “inflammatory macrophages” or “healing Tregs” might predict propensity for adverse remodeling) and into adjunct therapies to current MI care. For example, alongside reperfusion and standard post-MI medications, future patients might receive an immunomodulator that selectively boosts reparative immune cells or a fibroblast-targeted drug that improves scar architecture. While such interventions are not yet in clinical practice, the scientific groundwork is rapidly being laid by SCMO research.

### Heart failure and cardiomyopathy

Heart failure remains a leading cause of morbidity, mortality, and healthcare costs worldwide (Lesyuk et al., 2018). It often represents the end result of cumulative cardiac insults like ischemic, hypertensive, valvular, and others, and features myocardial fibrosis, cardiomyocyte hypertrophy or loss, microvascular rarefaction, and immune-cell infiltration (Zhang et al., 2024). The failing myocardium is a mosaic of regions with differing dysfunction and remodeling, and bulk profiling has historically obscured cell-specific alterations (He et al., 2025). Single-cell multi-omics now reveals how each cardiac cell type changes in HF and how those changes may be targeted (Zhang et al., 2024).

A landmark single-cell study of human dilated cardiomyopathy integrating tens of thousands of cells/nuclei showed a striking pattern: failing-heart cardiomyocytes converge on a shared “stressed” program (including fetal-gene re-expression), while non-myocytes diversify into HF-associated subpopulations absent in normal hearts (Koenig et al., 2022). Fibroblasts split into phenotypically distinct groups (e.g., proliferative, matrix-producing, inflammatory/developmental), macrophages into pro-inflammatory vs. pro-reparative states, and endothelial cells underwent broad transcriptional shifts related to angiogenesis and permeability without expansion of subtype number (Koenig et al., 2022). Therapeutically, this argues for cell-type-specific strategies—selectively targeting pathogenic fibroblast or macrophage states—alongside therapies aimed at shared cardiomyocyte failure pathways (e.g., metabolism, calcium handling), complementing foundational treatments such as beta-blockers.

Multi-omic profiling is also uncovering novel drivers and repurposing opportunities. In atrial fibrillation, single-nucleus multi-omics implicated androgen-receptor signaling in cardiomyocytes and NR4A1 regulation in fibroblasts, suggesting hormonal modulators as potential interventions in atrial cardiomyopathy (Leblanc et al., 2025).

From a diagnostic and prognostic perspective, SCMO-derived gene signatures may enable risk stratification and therapy selection. Machine-learning “scores” distinguishing healthy vs. failing cellular states could be applied to myocardial biopsies; circulating immune or fibroblast-like cells might offer less-invasive surrogates. These applications remain investigational, but they point toward precision cardiology that classifies HF by dominant cellular programs (e.g., inflammation-vs. fibrosis-driven) to guide tailored treatment.

### SCMO for longevity medicine

**FIGURE 1:**
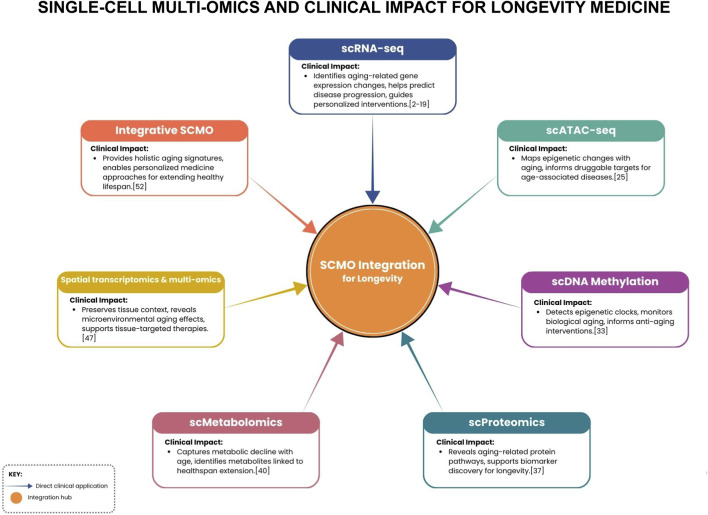
Schematic linking SCMO modalities to key clinical impacts for longevity, centered on an integration hub that fuses data to enable personalized interventions.

Aging stands as the foremost risk factor for cardiovascular and most chronic diseases (Kennedy et al., 2014). A recent study noted that certain transcriptional signatures in cells were associated with aging versus with HF, and some cell states seen in aged hearts overlapped with those in failing hearts. This suggests that the aging process primes some of the cellular changes that manifest pathologically in HF, a reminder that longevity and cardiovascular disease are intertwined at the cellular level (Koenig et al., 2022). Precision geromedicine (or longevity medicine) extends beyond simply increasing lifespan to maximizing healthspan, the years lived free of disability, by targeting actionable hallmarks of aging, including the overactivation of “gerogenes” that promote biological aging and the inactivation of “gerosuppressors” that normally slow the process. This approach leverages multi-omic and clinical biomarkers for early detection of age-related dysregulation, personalizing interventions (pharmacological, lifestyle, and psychosocial) to each individual’s unique molecular and clinical profile, with the goal of preventing or delaying the onset of age-associated pathologies (Kennedy et al., 2014; Kroemer et al., 2025) (Figure 1). SCMO aligns perfectly with these goals, as it can sensitively detect subtle molecular shifts in cells that precede overt disease and can capture the multifaceted effects of interventions (diet, exercise, drugs) at a cellular level.

### Early detection and biomarkers

Researchers are now using single-cell data to build “aging clocks” that can estimate an individual’s biological age with greater accuracy than chronological age. One study performed scRNA-seq on blood immune cells from individuals across the human lifespan, defining an “immune age” metric based on composite gene expression patterns (Wang Y. et al., 2025). This metric could flag early signs of immune dysfunction, such as the loss of naïve T-cells, in ostensibly healthy individuals, offering a potential window for preemptive intervention. Studies of supercentenarians (individuals over 110 years old) have provided further insights, revealing a unique and marked expansion of cytotoxic CD4^+^ T-cells (Hashimoto et al., 2019). This distinctive cellular profile may represent a critical immune adaptation that contributes to exceptional longevity by enhancing resistance to infections and malignancies. SCMO can also detect “priming” events that precede functional decline. For example, a multimodal single-cell analysis of aging human skeletal muscle showed that key stress-related genes become epigenetically primed, with their chromatin becoming more accessible, years before their expression levels actually increase. Such early molecular warning signs could be used as biomarkers to predict future sarcopenia and prompt earlier intervention with exercise or nutritional support (Lai et al., 2024).

### Personalized treatment and pharmacogenomics

Just as no two individuals have the same genetic code, the hallmarks of ageing manifest with considerable variation across individuals, influencing cellular composition and treatment responses (López-Otín et al., 2013). One aspect is classic pharmacogenomics, enhanced by single-cell resolution via sc-eQTL mapping that links genotype to cell- and state-specific expression effects (Nathan et al., 2022; van der Wijst et al., 2018). As described previously, SCMO allows us to personalize treatments by aligning them with a patient’s unique cellular makeup. One aspect is classic pharmacogenomics, which uses genomic data to predict drug response, but is enhanced by single-cell resolution. For instance, while genomic analyses have identified gene variants that modulate the efficacy of drugs like aspirin, SCMO could take this a step further by showing the downstream effect in the relevant cells—for example, confirming that a patient’s platelets are exhibiting the expected transcriptomic response to the drug (Lewis et al., 2020; Backman et al., 2017). In essence, a subgroup of patients (defined by genotype) has platelets or vascular cells that respond especially well to aspirin’s mechanism. In the future, a clinician could use an SCMO assay to not only genotype such variants in a patient but also see the downstream effect in relevant cells (e.g., are the patient’s platelets actually exhibiting the expected transcriptomic response to aspirin?). The landmark CANTOS trial demonstrated that the anti-inflammatory drug canakinumab reduced cardiovascular events, but only in a subset of patients (R et al., 2018; Baylis et al., 2017). In the future, single-cell profiling of a patient’s circulating immune cells could identify those with a specific “inflamed” profile, making them ideal candidates for such a therapy. This allows for the personalization of treatment by aligning it with a patient’s unique cellular makeup.

### Prevention and lifestyle interventions

Perhaps one of the most exciting Frontier for SCMO is its use in rigorously testing and monitoring preventative interventions, from exercise and diet to nutritional supplements. It is well established that exercise improves cardiovascular health, but individual responses vary widely (Sarzynski et al., 2022). Single-cell studies are beginning to decipher these differences (Rubenstein et al., 2022). A recent single-cell atlas of ageing human skeletal muscle revealed transcriptional programs in mesenchymal and muscle stem cells relevant to tissue remodeling, providing insights into how exercise might act through specific cell populations (Lai et al., 2024). This provides a detailed cellular map of how exercise confers its benefits. For longevity medicine, this means we can move beyond generic recommendations. A clinician could use single-cell analysis of a patient’s blood or tissue to objectively monitor whether a new exercise regimen is activating the desired beneficial cell programs. If not, the type or intensity of the exercise could be adjusted in a data-driven manner. Similarly, single-cell studies of exercise interventions already demonstrate measurable cellular remodeling (Rubenstein et al., 2022; Ruffino et al., 2016), and comparable approaches could in principle be extended to other lifestyle strategies, helping to evaluate which regimens truly produce beneficial molecular changes.

An emerging concept is the “digital twin” for aging, a computational model of a patient that can predict how they would respond to various interventions (Björnsson et al., 2020). SCMO provides the granular data needed to build such models. If one has SCMO data from a patient (say, their immune cell profiles, skin cell epigenetic age, etc.), one could computationally simulate the effect of a drug or lifestyle change on those profiles (leveraging large databases of how those interventions affected similar cells in research studies) (Lotfollahi et al., 2023). While still futuristic, this kind of AI-driven approach may guide personalized longevity plans, for example, predicting that Patient A’s cells would benefit more from a senolytic drug (because they have a high burden of senescent-cell signatures), whereas Patient B’s profile suggests more benefit from mitochondrial antioxidants. The integration of SCMO with advanced analytics and AI represents a crucial Frontier for precision prevention and longevity medicine.

Table 1 summarizes recent single-cell multi-omics findings across cardiovascular and aging contexts and links key cell states to potential clinical uses.

**TABLE 1:** Overview of clinically relevant findings derived from recent SCMO research across various disease areas.

Disease area	Key SCMO findings	Cell types/Molecular targets identified	Potential clinical applications	Key translational caveat
Atherosclerosis/CAD	Immune heterogeneity in plaques; SMC phenotypic modulation in advanced disease (Wirka et al., 2019; Mosquera et al., 2023; Fernandez et al., 2019; Depuydt et al., 2020; Lin et al., 2024; Ye et al., 2025)	TREM2^hi foamy macrophages; CSF1-responsive foamy macrophages; Treg subsets; LTBP1^+^/CRTAC1^+^ SMC states; multi-lineage SMC_C5 (Mosquera et al., 2023; Xiong et al., 2023; Fernandez et al., 2019; Depuydt et al., 2020; Ye et al., 2025)	Risk stratification via immune-state signatures; targeted anti-inflammatory therapy (IL-1β blockade, colchicine) (Xiong et al., 2023; Lin et al., 2024; Nidorf et al., 2020; Ridker et al., 2017; R et al., 2018)	Targeting immune subsets requires high-specificity approaches to avoid systemic immunosuppression
Myocardial Infarction	Temporal dynamics of immune and fibroblast subtypes after injury (scRNA-seq & spatial/multi-omics) (Kuppe et al., 2022; Vafadarnejad et al., 2020; Farbehi et al., 2019)	IRF3-driven macrophages; CKAP4^+ fibroblasts; B2M-linked signaling; Wif1^+ fibroblasts; SPARC^+ Tregs (mouse) (Molenaar et al., 2021; King et al., 2017; Xia et al., 2020; Wang et al., 2023; Gladka et al., 2018; Farbehi et al., 2019)	Targeted anti-inflammatory or anti-fibrotic strategies; cell-state-based prognostic tools (Kuppe et al., 2022; Molenaar et al., 2021; King et al., 2017; Vafadarnejad et al., 2020; Gladka et al., 2018; Farbehi et al., 2019)	Analytical turnaround times are currently incompatible with acute diagnostic/therapeutic windows
Heart Failure	Cardiomyocyte convergence vs. stromal/immune diversification; fibroblast/myeloid diversification (Zhang et al., 2024; He et al., 2025; Koenig et al., 2022)	Proliferative/inflammatory fibroblast and divergent macrophage states; AR signaling occurs in AF rather than HF (Zhang et al., 2024; Koenig et al., 2022; Leblanc et al., 2025)	Subtype-guided therapy (fibrosis- vs. immune-dominant HF) (Zhang et al., 2024; Koenig et al., 2022)	Requires invasive myocardial biopsies; less-invasive surrogates are not yet validated
Aging/Longevity	Single-cell aging clocks and immune remodeling; early epigenetic shifts (single-cell DNAm) (Zhu et al., 2023; Wang Y et al., 2025; Lai et al., 2024; Bell et al., 2019; Bonder et al., 2024)	Cytotoxic CD4^+^ T-cell expansion in extreme age; naïve↔memory T-cell ratio remodeling; PBMC ribosome↔inflammation balance/AP-1 priming (Zhu et al., 2023; Wang Y et al., 2025; Hashimoto et al., 2019)	Biological-age diagnostics; precision geromedicine frameworks (Kroemer et al., 2025; Bell et al., 2019; Bonder et al., 2024)	Clocks remain research tools; clinical utility and impact on outcomes are unproven
Oncology	Detection of rare malignant and immune subclones with single-cell methods (Lei et al., 2021; Ally and Chen, 2024)	MRD clones; T-cell receptor signatures (Lei et al., 2021; Ally and Chen, 2024)	Early relapse prediction; immunotherapy monitoring (Lei et al., 2021; Ally and Chen, 2024)	Detecting very rare cells requires high input and optimized pipelines; scalability is challenging
Pharmacogenomics	Cell-type-specific genotype→phenotype links (sc-eQTL); perturbation screens (Nathan et al., 2022; van der Wijst et al., 2018; Lotfollahi et al., 2023)	Pharmacogene variability across cell types (Nathan et al., 2022; van der Wijst et al., 2018)	Responder stratification (CANTOS; aspirin pharmacogenomics); sc-perturbation models (Ridker et al., 2017; R et al., 2018; Lewis et al., 2020; Backman et al., 2017; Lotfollahi et al., 2023)	Actionable decision rules from single-cell readouts are not established for most therapies
Lifestyle Medicine	Cell-type-specific responses to exercise/caloric restriction; monocyte PPARγ programs; muscle aging atlas (Lai et al., 2024; Sarzynski et al., 2022; Rubenstein et al., 2022; Ruffino et al., 2016)	Mesenchymal/stem-cell niches and immune compartments shift with age in skeletal muscle (Lai et al., 2024)	Objective monitoring of interventions; digital twins/predictive models; personalized healthspan optimization (Kroemer et al., 2025; Björnsson et al., 2020; Lotfollahi et al., 2023)	Costs and sampling burden limit routine use; validated cellular ‘success signatures’ are pending

Specific cell types and molecular targets identified through SCMO analyses are listed, alongside potential near-term clinical applications. These insights illustrate the practical utility of SCMO for enhancing diagnostics, improving patient risk stratification, guiding targeted therapeutic strategies, and monitoring the effectiveness of lifestyle and pharmacological interventions. Abbreviations: HF, heart failure; AML, acute myeloid leukemia; MRD, minimal residual disease; CR, caloric restriction; Treg, regulatory T cell; AR, androgen receptor.

### Translational outlook: from bench to bedside

An increasing number of clinical trials, particularly in oncology, are incorporating single-cell analyses as exploratory endpoints to stratify patients, monitor treatment responses, and understand mechanisms of drug resistance (Lei et al., 2021). This model is expected to be adopted in cardiology and gerontology trials. For instance, a heart failure drug trial might include optional endomyocardial biopsies for single-cell analysis to provide molecular evidence of whether the drug is shifting cardiac cell states in the desired direction, accelerating translational learning. Another key avenue to clinical practice is the development of SCMO-based diagnostic tools. While a CLIA-approved “single-cell test” is not yet a reality, progress is being made on liquid biopsy techniques that isolate and analyze individual circulating cells for diagnostic purposes in oncology and immunology (Ally and Chen, 2024; Kante et al., 2025). In cardiovascular disease, one can envision a blood test that performs multi-omic analysis on circulating immune cells to detect a pro-atherogenic signature long before a plaque causes symptoms. Foundational to this progress are large-scale reference mapping projects like the Human Cell Atlas, which are compiling comprehensive maps of all human cell types (Kante et al., 2025; Litviňuková et al., 2020). These atlases will serve as essential baselines against which clinical samples can be compared, facilitating the interpretation of patient data. For example, if a certain macrophage subtype is overrepresented in a myocarditis patient compared to the healthy heart atlas, it might suggest a targeted anti-inflammatory therapy. As these atlases become publicly available resources, they will facilitate the clinical interpretation of single-cell data, serving as an important bridge to real-world use. In cardiovascular disease, spatial multi-omics could help identify microscopic regions of active inflammation in myocarditis or transplant rejection biopsies earlier than histology can (Amancherla et al., 2025; Mantri et al., 2022).

As SCMO enters practice, clinicians will need tools to help make sense of the high-dimensional data. In the near-term, the most likely scenario for clinical SCMO is as a complementary tool in specialized cases. Within the next several years, we might see academic medical centers offering a “single-cell sequencing analysis” of certain biopsies or blood samples as a send-out test, primarily for complex cases that do not fit typical patterns. Cardiologists may collaborate with research labs to sequence a cardiac biopsy from a patient with unexplained cardiomyopathy to identify molecular clues (for example, is there an upregulation of viral response genes, suggesting myocarditis?). As data accumulates and these experiences get published, the utility will become clearer and the practice could spread.

In summary, the outlook for SCMO in clinical practice is bright but requires continued innovation and validation. The next few years are expected to yield the first wave of clinically useful single-cell tests (especially in oncology and immunology), and cardiovascular/longevity applications will follow as evidence builds. Given the richness of information SCMO provides, it has the potential to transform how we classify diseases, like subtyping heart failure patients by the cellular makeup of their hearts, and how we choose therapies. The ongoing challenge is translating that richness into something actionable and accessible for everyday clinical use, a challenge the field is actively addressing through better technology, reference atlases, and integrative analytical tools.

For quick reference, Table 2 lists where SCMO has been applied directly to patient samples and what was learned.

**TABLE 2:** Key Clinical Implementations of Single-Cell Multi-Omics in Human Samples. Study-level register of SCMO applied directly to human/clinical specimens; for each clinical context, it lists the cited study, patient material/cohort, multi-omic modality, and the main translational readouts.

Clinical context	Study (citation)	Patient material/Cohort	SCMO approach	Clinical/Translational readouts
Atherosclerosis (plaque immunology)	Nat Med (Fernandez et al., 2019)	Human atherosclerotic plaques (immune cells)	CITE-seq (RNA + surface proteins)	Discriminated macrophage and immune subsets; refined plaque immune heterogeneity readouts
Coronary syndromes (ACS/CCS)	Nat Med (Pekayvaz et al., 2024)	Clinical cohorts with acute and chronic coronary syndromes	Multi-omic immune profiling	Identified immunological signatures associated with ACS/CCS; feasibility of multi-omic immune readouts in cardiology cohorts
Myocardial infarction (post-MI human hearts)	Nature (Kuppe et al., 2022)	Human myocardial tissue from patients with prior MI	snRNA-seq + snATAC-seq (multiome; spatial mapping)	Linked expression to chromatin; RUNX1 highlighted as a candidate master regulator of fibroblast→myofibroblast differentiation
Atrial fibrillation	Nat Cardiovasc Res (Leblanc et al., 2025)	Human atrial tissue	Single-nucleus multi-omics	Implicated androgen-receptor signaling (cardiomyocytes) and NR4A1 regulation (fibroblasts), suggesting pathway-level targets
Cardiac transplant rejection	Amancherla et al. (2025)	Human heart allografts (transplant setting)	Spatial transcriptomics	Resolved dynamic rejection-related programs *in situ*; demonstrates biopsy-adjacent spatial omics in a clinical context
Viral myocarditis	Nat Cardiovasc Res (Mantri et al., 2022)	Myocarditis tissue	Spatiotemporal transcriptomics	Resolved pathogenic trajectories in myocarditis using spatial/temporal single-cell methods
Aging/skeletal muscle	Nature (Lai et al., 2024)	Human skeletal muscle across age	Multimodal atlas (single-cell/single-nucleus)	Defined age-associated cellular programs relevant to longevity medicine and intervention monitoring

## Challenges in integration strategies and analytical tools

Aging is a multifaceted process, governed by the interplay of intrinsic genetic determinants and extrinsic environmental factors, which necessitates the multimodal approach detailed in this manuscript. This is compounded by the fact that the aging process has a stochastic element: not only do people age at different rates, but aging progresses differently across cell types within the same individual (He et al., 2020; Martin, 2012; Crane et al., 2020). Although aging is universal, its complexity and variability demand especially sophisticated analytical approaches.

Single-cell sequencing data analysis shares similarities with bulk sequencing analysis, yet it presents distinct challenges. For instance, bulk sequencing averages gene expression across numerous cells to provide a homogenous profile, whereas single-cell sequencing yields sparse gene-count matrices characterized by a high proportion of null values (Yu et al., 2021; Stegle et al., 2015). Therefore, the statistics employed for single-cell sequencing analysis should be different from those employed for bulk analysis, and are required to handle a large number of missing values, the alternative being the imputation of said values (Hicks et al., 2018; Chen et al., 2019).

Normalization methods also present a significant challenge, particularly given the expected variability in gene expression both between and within cell types. Consequently, the normalization of gene expression levels necessitates careful consideration (Luecken and Theis, 2019). The analysis of single-cell sequencing data has led to the development of numerous algorithms, approximately 1700, according to a recent study, yet the field is still developing. Consequently, many commonly employed tools and filtering thresholds may lack optimization or universality (Luecken et al., 2024; Arcos Hodar et al., 2024). This compounds the challenges facing the study of aging and longevity: for example, the scientists working on the cell rejuvenation atlas reported inconsistent filtering thresholds for low-quality cells across three major atlases of calorie restriction (Arcos Hodar et al., 2024).

The integration of data derived from diverse analysis types and platforms presents a further impediment. Given that aging is correlated with an elevated incidence of single-nucleotide variants, alongside more intricate genomic events such as insertions, deletions, and copy number variations, the concurrent analysis of RNA and DNA from the same cell makes biological sense (Ren et al., 2022; Lodato et al., 2018). At the same time, global and local changes in DNA methylation patterns are well documented during aging, warranting bisulfite DNA sequencing (López-Otín et al., 2013; Bell et al., 2019; Bonder et al., 2024). The scarcity of longitudinal studies on aging at the single-cell level is another significant consideration. While numerous algorithms have been designed to integrate these multifaceted datasets, they encounter substantial challenges (Ma et al., 2020).

## Limitations

### High cost and technical complexity

Single-cell multi-omic assays are currently expensive, both in terms of reagents and the required instrumentation (Wu et al., 2024). Analyzing thousands of single cells with multiple-omic layers can easily run into the thousands of dollars per sample (Wu et al., 2024; Minoshima et al., 2021). This high cost restricts the size of studies and prevents large clinical validation cohorts. As a result, most SCMO studies to date have focused on discovery in relatively small sample sets, with any validation performed using less expensive methods (such as bulk PCR or immunostaining). For clinicians, this means we do not yet have the level of evidence, from large, multi-center trials, that would be ideal for incorporating SCMO-based biomarkers into guidelines.

The complexity of experiments is another barrier, isolating viable single cells or nuclei from clinical tissue can be complicated. Many clinical tissues (e.g., calcified plaques or fibrotic hearts) are difficult to dissociate into single cells. Moreover, some cell types do not survive the processing well. Strict sample requirements (often fresh tissue, rapid processing) limit SCMO’s immediate applicability in routine pathology labs (Bakken et al., 2018). If a sample has to be sent to a specialized center and processed within hours of collection, that limits throughput and accessibility. Encouragingly, methods are improving, for example, single-nucleus sequencing allows the use of frozen samples (nuclei can be extracted from stored tissue), and there’s progress in integrating tissue-preservation protocols with single-cell analysis.

### Data volume and interpretability

Single-cell multi-omics experiments generate extensive datasets, comprising tens of thousands of cells, each characterized by thousands of molecular features. Interpreting such large datasets poses a significant challenge, necessitating specialized computational methods to extract biologically or clinically relevant information (Hao et al., 2021). The limited availability of bioinformatics experts in typical hospital laboratories raises concerns about interpretability. Translating single-cell data into actionable reports for treating physicians often requires collaboration with research teams. Although efforts are underway to develop simplified metrics, such as cellular inflammation scores or fibrosis indices, consensus on these metrics is still lacking. Furthermore, the complexity of the analysis introduces variability, as different bioinformatics pipelines may yield varying results from the same raw data. The absence of standardized analysis workflows, potentially validated by regulatory bodies, raises concerns about inconsistent interpretation across different centers.

### Standardization gaps

Since SCMO is so cutting-edge, there is currently no uniform playbook that all labs follow. Different protocols may employ different chemistries (such as which genes are captured), and various analysis methods can differ in how they identify cell types or gene programs. This complicates comparison across studies and is a major issue for clinical deployment, where standardization is key. For example, in single-cell immune repertoire sequencing, some methods capture only one chain of the T-cell receptor, or occasionally two cells might get mixed (“doublets”), leading to incorrect receptor pairing (Xi and Li, 2021). These technical artifacts can confound results. While computational tools exist to detect and filter out such artifacts, they are not foolproof. The community will need to agree on best practices for quality control, such as, what percentage of doublets is acceptable, how to validate that a given cell cluster is real and not an artifact. In clinical labs, assays typically undergo rigorous validation and have quality metrics; SCMO will need the equivalent. Efforts like the Human Cell Atlas are helping by providing reference standards (Regev et al., 2017). Until standardization improves, results from SCMO must be interpreted with caution, especially if making high-stakes clinical decisions.

### Analytical challenges and integrating multi-modal data

By its nature, multi-omics means dealing with different data types simultaneously (DNA mutations, RNA levels, protein abundance, chromatin marks, etc.). Integrating these layers is challenging–sometimes the layers can give seemingly conflicting information. For example, a cell might have an open chromatin mark at a gene (suggesting it could express that gene) but low RNA expression of it, due to post-transcriptional regulation. Deciding how to weigh each layer in interpreting cell state requires sophisticated analysis and sometimes new computational frameworks. If not done properly, one might draw wrong conclusions (e.g., overestimating the importance of a mutation that is not actually expressed at RNA/protein level). The development of robust computational tools for data integration is an active area of bioinformatics, and until they mature, multi-omic data interpretation will be a job for specialists. Clinicians will likely rely on summary outputs and visualizations rather than raw data, but they should be aware that behind that simplicity is a complex analysis with assumptions and statistical uncertainty. This is analogous to how complex algorithms underlie an ejection fraction measurement on cardiac MRI–the clinician sees the number but might not know all the math behind it. Transparency and validation of these analytic methods will be crucial for trust in SCMO-based tests.

### Clinical utility and validation

Perhaps the biggest question: does SCMO improve patient outcomes enough to justify its complexity and cost? This remains to be proven in most domains. As one commentary has pointed out, the incremental benefit of single-cell resolution over bulk for clinical decision-making remains uncertain in many cases (Kim et al., 2021). For example, if bulk gene expression from a biopsy already tells us a tumor is EGFR-positive, do we need single-cell data to guide therapy? Possibly not in that case. The answer will vary by scenario. Single-cell data may be particularly important when heterogeneity itself is clinically significant (e.g., cancer resistance clones, immune cell subtypes in a granuloma), but less critical when a tissue is relatively uniform or when bulk signals are sufficient. So, we need studies that directly compare using SCMO informed care vs. standard care. These might be trials where one arm receives a therapy guided by a single-cell biomarker and another arm receives a therapy guided by conventional markers, to see if outcomes differ. Until such trials are done, many payers will be reluctant to cover these expensive tests, and guidelines will be cautious in endorsing them. In short, SCMO must demonstrate its clinical utility in an evidence-based fashion. Early hints are promising as reported in previous sections, but moving from correlation to actual patient benefit is the next step.

While FDA-authorized single-cell diagnostics do exist, to the best of our knowledge, as of 2025 they are exclusively limited to established technologies like flow cytometry for immunophenotyping. The next-generation of single-cell multi-omic tools, which provide far deeper genomic and transcriptomic data, have not yet crossed the regulatory threshold into routine care and remain confined to the research domain. Companies label their single-cell assay kits as “for Research Use Only,” and any clinical application presently occurs under the scope of research or LDTs. We mention this to set realistic expectations: clinicians reading about these exciting discoveries should understand that they are not yet available as ready-to-use clinical tests for patient care. However, we also note that some academic centers have started implementing single-cell assays in clinical research studies (for example, deep immune profiling of patient blood samples within a trial), a step toward eventual routine use.

### Ethical and logistical considerations

The substantial depth SCMO data introduces considerable ethical and logistical responsibilities. The capacity to resolve cellular heterogeneity means that patient-derived datasets may uncover incidental findings, such as rare pathogenic mutations present in somatic clones. This potential raises complex ethical questions regarding the disclosure of such findings and the subsequent requirements for genetic counseling. Furthermore, the vast scale and inherent identifiability of genomic information present significant data privacy challenges. Consequently, the clinical implementation of SCMO necessitates the development of rigorous frameworks for informed patient consent and secure data governance. Finally, a critical limitation is the analytical turnaround time. The current workflow, from sample acquisition to final bioinformatic result, can span several days to weeks, a timeframe often incompatible with the decision-making windows in acute care settings. In parallel, research efforts are actively seeking to accelerate this pipeline. The integration of emerging technologies, such as microfluidics and real-time on-chip sequencing, holds promise for near-patient applications, although these solutions are not yet mature for routine clinical practice.

## Conclusion

Single-cell multi-omics has opened a new Frontier in medicine, providing a lens of unprecedented resolution through which to view the cellular and molecular basis of cardiovascular aging and disease. By deconstructing complex tissues into their constituent parts, SCMO is transforming our understanding of pathologies like atherosclerosis, myocardial infarction, and heart failure, revealing them not as uniform entities, but as heterogeneous and dynamic ecosystems of interacting cells. The discoveries highlighted in this review, from pathogenic T-cell subsets that predict plaque rupture to reparative macrophage populations that dictate the quality of post-infarction healing, are not merely academic curiosities. They point toward a new generation of precision diagnostics and targeted therapeutics.

However, it is crucial to maintain a perspective of engaged skepticism. While the scientific potential of SCMO is immense, significant barriers related to cost, methodological complexity, and data interpretation currently limit its accessibility and clinical reliability. What remains critical is bridging the gap between bench and bedside: multi-center validation, simplification of workflows, and demonstrating that SCMO-based decisions improve patient outcomes. If these can be achieved, single-cell multi-omics will probably become a key pillar of precision medicine in the era of aging populations.

The scientific community is actively engaged in surmounting these obstacles through technological innovation, the development of robust analytical methods, and the establishment of large-scale collaborative projects to ensure reproducibility. Economic barriers are diminishing rapidly; for context, the cost of sequencing the first human genome was approximately $3 billion, whereas contemporary single-cell transcriptomic analyses represent a minute fraction of this initial investment.

For the clinical community, the current landscape calls for cautious optimism. Clinicians should be aware of the limitations when evaluating SCMO-based research but should also recognize its transformative potential. As these challenges are progressively overcome, SCMO is poised to become a foundational pillar of precision medicine. In the coming decade, we can anticipate the emergence of clinical tests that subtype a patient’s heart failure based on the cellular composition of their myocardium, or that stratify their risk of a heart attack based on the inflammatory signature of their circulating immune cells. Until that time, SCMO should be regarded as a powerful, specialized tool to be utilized primarily as an adjunct to, rather than a replacement for, established clinical data and diagnostic assessments.
